# Photon density wave spectroscopy as process analytical technology: a review of recent advances in technology and application

**DOI:** 10.1007/s00216-025-06200-8

**Published:** 2025-11-06

**Authors:** Thomas Schiewe, Aaron Justin Koenig, Björn Weiske, Roland Hass

**Affiliations:** 1 Chair of Bioprocess Engineering, Institute of Biotechnology, Technische Universität Berlin, Berlin, Germany; 2 innoFSPEC Transfer Lab, Institute of Chemistry, University of Potsdam, Potsdam, Germany; 3 Corden BioChem International GmbH, Brüningstr. 50, 65929 Frankfurt, Germany; 4 PDW Analytics GmbH, Geiselbergstraße. 4, 14476 Potsdam, Germany

**Keywords:** PDW spectroscopy, PAT, Particle sizing, Inline, Emulsions, Suspensions, Fermentation

## Abstract

Photon density wave (PDW) spectroscopy is a calibration-free method for the simultaneous, absolute, and independent quantification of the optical absorption coefficient and the reduced scattering coefficient of highly light scattering materials. It allows for dilution-free particle sizing in concentrated liquid systems. Thus, it is beneficial as an inline process analytical technology (PAT). A brief comparison to other particle sizing technologies, the theoretical background of PDW spectroscopy, the current technical status, and limitations and technical challenges are reviewed. In addition, a comparative overview of the recent applications of PDW spectroscopy as PAT for chemical, biotechnological, and food processing is provided.

## Introduction

Photon density wave (PDW) spectroscopy [1–7] yields the reduced scattering coefficient (*μ*_s_′), the absorption coefficient (*μ*_a_), and the particle size from concentrated, highly turbid heterophase systems, i.e., emulsions and suspensions. These parameters are obtained in real time with high temporal resolution and without dilution of the liquid dispersions. Together with a probe-based implementation into reaction vessels, these outcomes make PDW spectroscopy relevant for the utilization as process analytical technology (PAT) in industry segments like food, chemistry, pharmaceuticals, and biotechnology.

**Fig. 1:**
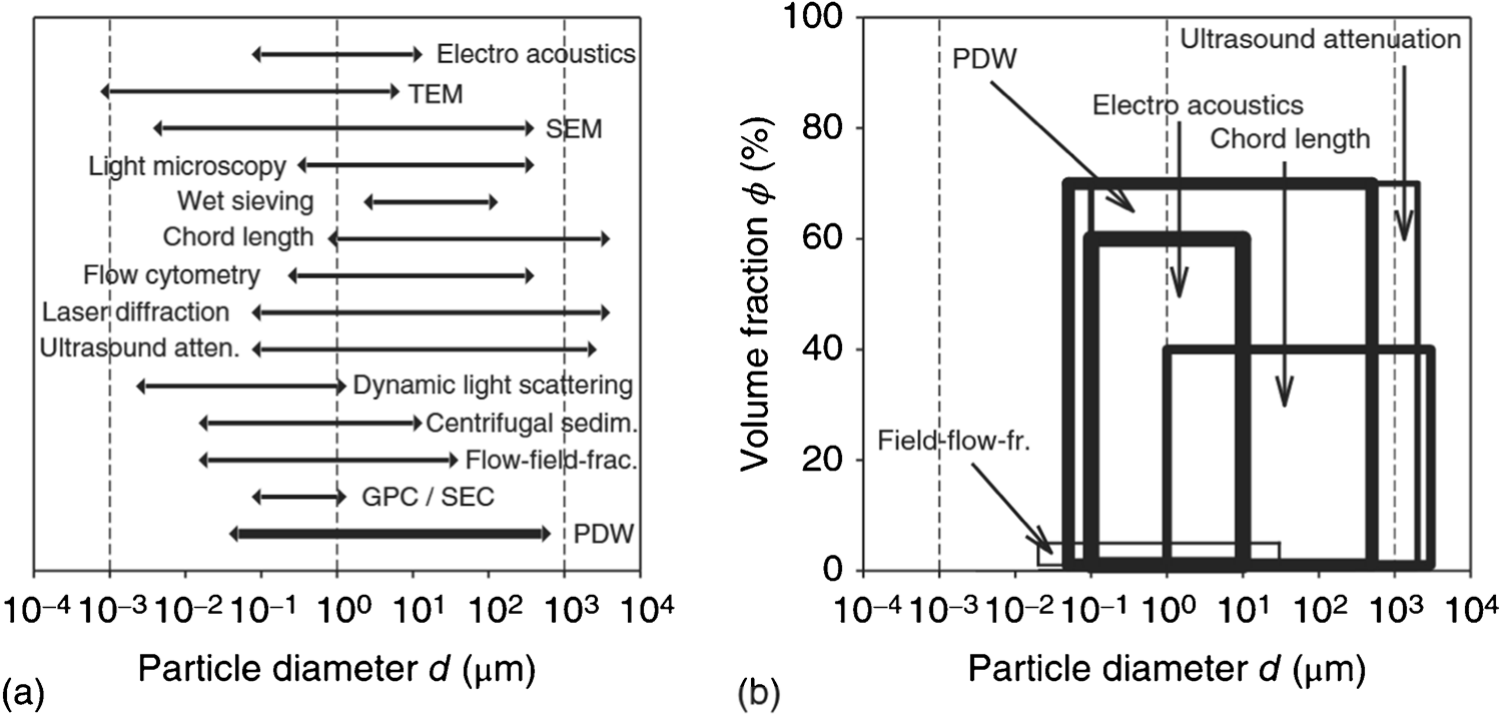
Sizing ranges (**a**) and volume fraction ranges (**b**) for common particle sizing methods. Data are generalized and need to be verified for a defined material under investigation. Based on [8], range for PDW spectroscopy added, figures from [9]

While the optical coefficients are often already extremely meaningful for process monitoring and control, e.g., for batch to batch consistency, the main interest in the PDW technology is found in its capability for inline particle sizing. Fundamentally, particle sizing needs to be differentiated between single particle characterization (e.g., sizing in electron microscope images) and particle ensemble characterization, where a multitude of particles simultaneously contribute to the analytical signal (e.g., static light scattering). PDW spectroscopy is an ensemble method. The virtue of single particle characterization is obvious: it is the best approach towards particle size histograms. Single particle characterization, however, becomes more and more challenging when concentrated, industrially relevant dispersions are encountered. In practice, sizing for concentrated samples often requires sampling and dilution. However, ensemble methods may also face upper concentration limits and dilution requirements. In addition, ensemble methods require the use of assumed size distribution functions like Gaussian or log-normal. Without claiming completeness and for all existing particle systems, Fig. 1 displays size and volume fraction ranges for particle characterization technologies including PDW spectroscopy.

PDW spectroscopy can typically measure particle diameters without dilution in the range from 50 nm to 500 µm and up to 70 vol%. It has to be clearly stated that these ranges do not hold for all particle systems, but depend strongly on the individual optical characteristics and thus need to be evaluated per material system. The virtues of PDW spectroscopy for inline particle sizing are applicability to concentrated systems, nano- and micrometer-scaled particles, systems under stirring or under flow and/or higher viscosity, time resolution in the sub-minute regime, limited issues with so-called process probe fouling, and data analysis towards particle size entirely based on theory. Its disadvantages are unsuitability to strongly absorbing suspensions, challenges for particle size distribution determination [10], challenges with too low turbidities, the need for the knowledge of refractive index as well as volume fraction [11], and the required further technical development, especially of the process probe concept (see ” for details).

PDW spectroscopy is a frequency-domain approach, also called frequency-domain photon migration (FDPM) by early important innovators in the field such as Tromberg et al. and Sevick-Muraca et al. [2, 12–19]. Other important groups were Fishkin, Fantini, Gratton et al. [1], without claim for completeness. In the late 1980 s to early 2000 s, several other groups were active in researching this promising new technique [5, 6, 20]. Parts of the theoretical background were further developed by Reich and Bressel [7, 21, 22]. From 2010, development activities were driven towards the application of PDW spectroscopy as PAT with a special focus on highly concentrated dispersions (see ”). Nowadays, the term FDPM is used more often in the area of biomedical instrumentation, whereas PDW spectroscopy is used in the area of chemistry, but may also be employed in other areas such as bioprocessing. However, for this review, no evidence has been found that FDPM has been used or further developed as a PAT in the process industry. As a complementary alternative, time-domain experiments can be executed to obtain the optical coefficients for heterophase materials [23–31]. Again, no evidence could be found that this complementary approach was or is used as PAT in the process industry.

The work on particle sizing presented here is based on earlier work by many authors in the field of scattering theory. Refer for example to the compiled works from Bohren and Huffman [32], who have extensively described the calculation of scattering parameters of particles based on Mie theory. The inverse problem, obtaining a particle size from scattering parameters, was described in detail by Kerker et al. [33]. Discussions about scattering and particle sizing in highly concentrated and charged systems are provided below.

Up to now, PDW spectroscopy has been applied for PAT mainly in academic and industrial R&D and pilot-scale reaction systems. Applications will be discussed in more detail below. As PAT, the most prominent published fields in recent years have been polymerization [34–43], emulsification [34, 44–46], crystallization/precipitation [9, 47–49], cell cultivation [50–53], and enzymatic conversion [54].

## Fundamentals of photon density wave spectroscopy

### Optical coefficients from PDW spectroscopy

The theoretical and technical background of PDW spectroscopy is extensively described in various publications, e.g., by Bressel et al. [7]. In brief, the method relies on laser light that is sinusoidally intensity modulated in the MHz to GHz range. The electrical modulation signal is generated by a vector network analyzer (VNA). The modulated laser light is guided into the sample via an optical fiber. Applied laser wavelengths range from 400 to 1000 nm, depending on the experimental needs.

**Fig. 2:**
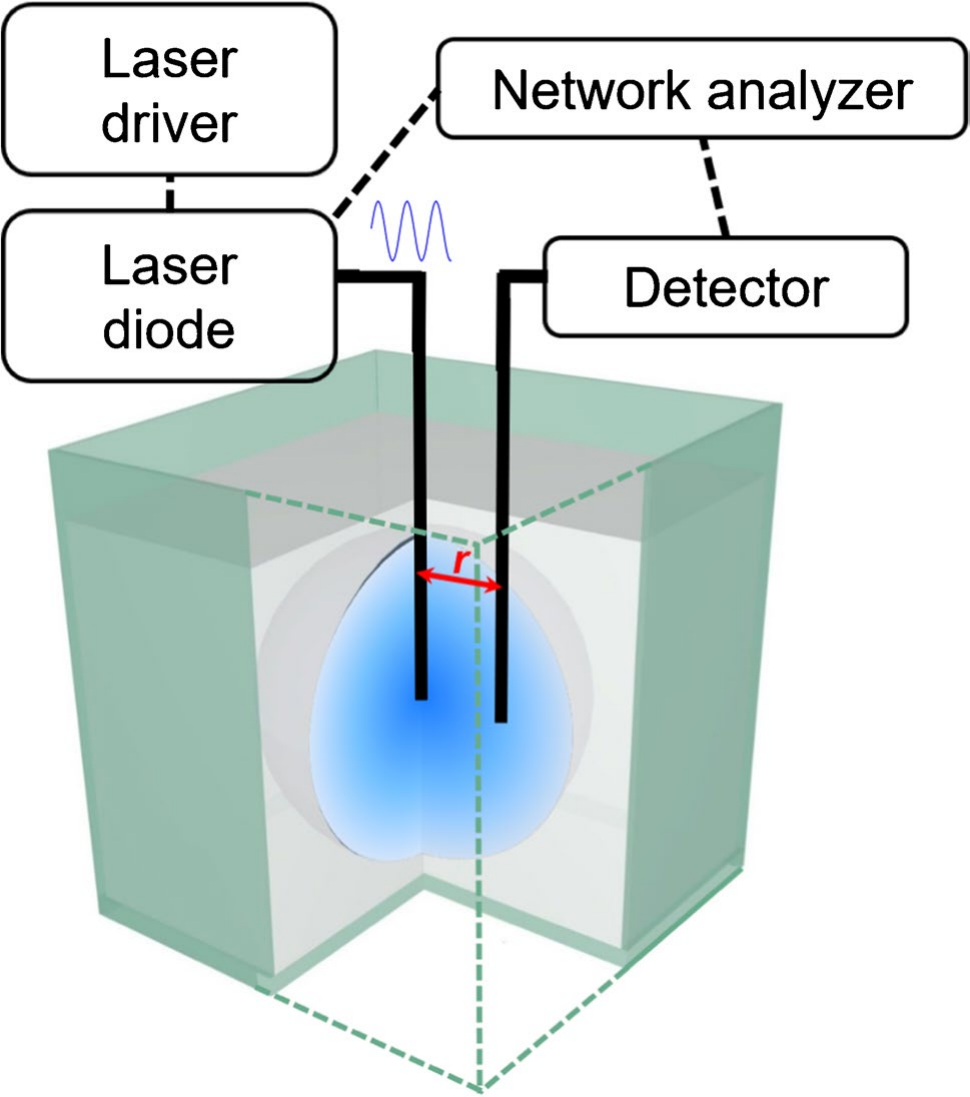
Schematic overview of the PDW spectroscopy experimental setup. The PDW (blue) propagates through the sample (grey volume) resulting from multiple scattering of the light emitted by the point-like light source at the fiber tip. The detector fiber at a distance *r* from the emission fiber guides light back to the detector. Intensity and phase changes of the PDW are characterized by the vector network analyzer. Adapted from [56]

Hass and Reich [34] describe the propagation of the laser light within the sample as follows: “[…] intensity-modulated laser light is guided via an optical fiber into the turbid material, where the fiber end acts as a point light source. The laser light interacts via absorption and multiple scattering with the dispersion.” Based on this interaction, a PDW is created. “It can be understood as a periodically changing number of photons per volume which, assuming a point-like light source, expands spherically” [55]. The experimental setup is schematically shown in Fig. 2.

**Fig. 3:**
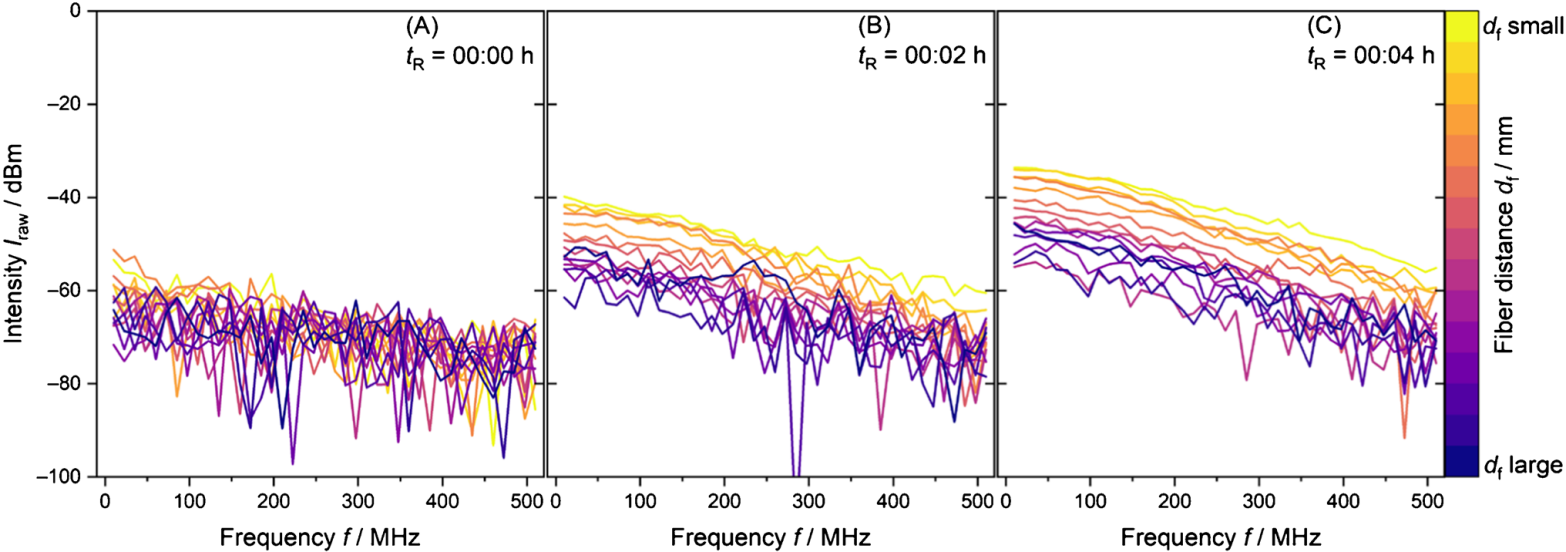
PDW spectroscopy raw data measurements at 638 nm of a vinyl acetate/Versa® 10 copolymerization before addition of monomer mixture (**A**), 2 min after addition (**B**), and 4 min after addition of the monomer (**C**) [43]

With variation of the detection fiber distance *r* to the emission fiber, photons from the PDW are collected and guided to a detector. The resultant electrical signal is processed by the VNA, whose output is referred to as the raw signal, representing intensity and phase as a function of modulation frequency and fiber distance. As an example, Schlappa et al. [43] showed the changes in raw intensity signal over the course of a vinyl acetate (VAc)/Versa® 10 copolymerization (Fig. 3, phase signal not shown). For this system and spectrometer, intensities below −60 dBm represent measurement noise. With an increasing scatterer content, the magnitude of light scattering increases, which results in a change from measurement noise (Fig. 3A) to increasing intensities (Fig. 3B and C) of detected modulated light at each fiber distance.

Such a “measurement set” of intensity and phase data includes the raw signal for all predefined measurement frequencies and fiber distances per temporal data point and per experimental wavelength. This measurement set is then processed into further raw data analysis and analysis towards the optical coefficients. The optical coefficients are obtained on the basis of radiation transport theory by a weighted two-dimensional multiple nonlinear global analysis of the raw intensity and phase data [57–59].

To obtain valid optical coefficients from PDW spectroscopy, three conditions must be met. Scattering of light must be much stronger than light absorption (*μ*_s_′≫ *μ*_a_), the sample volume must be large enough so that boundary losses of measurement light may be neglected, and light must scatter multiple times in the sample [1]. The lower boundary of the reduced scattering coefficient is material and experiment dependent. For a polystyrene (PS) dispersion, it was determined to be around *μ*_s_′ > 0.05 mm^−1^ [60]. The minimal sample volume depends on the optical coefficients and is often found to be 0.5 L or larger.

PDW spectroscopy is a calibration-free technique, as only the relative changes of the PDW as a function of fiber distance and modulation frequency are characterized. The resulting phase and intensity coefficients are linked to the optical coefficients by theory [1]. Thus, instrument factors, e.g., laser intensity, or system parameters like temperature, pH, viscosity, or pressure do not need to be considered or calibrated into data analysis. However, there are practical restrictions with respect to material compatibility or fabrication limits (see ” for details). The accuracy of the determined optical coefficients can be scrutinized by dilution series of the material under investigation. If the absorption properties of the continuous or dispersed phase are known, they can be compared to the results obtained from the dispersion by PDW spectroscopy [4, 46, 60]. For the scattering properties, the comparison to reference particle sizing indicates if the reduced scattering coefficients were obtained correctly [9, 46].

### Information from raw intensity data — PDraW

In case the above-mentioned boundary conditions are not met, PDW theory should not be applied for raw data analysis towards the optical coefficients. However, based on the raw data, mainly the detected intensities, useful information about the investigated system can still be obtained. For example, systems are encountered in which particle formation and thus turbidity generation occurs at a later stage during processing. As a consequence, the theoretical model of PDW spectroscopy for determining the optical coefficients cannot be applied initially. The actual spectrometer hardware, however, is extremely sensitive to changes of very low turbidities, i.e., even significantly below the lower threshold of PDW theory of *μ*_s_′ ≈ 0.05 mm^−1^. While data analysis for the optical coefficients below this threshold is unreliable, changes to the raw signal can be analyzed and yield meaningful turbidity process trends. This relative “PDraW” signal *I*_PDraW_ is calculated by integrating the detected intensities (Eq. 1):

1 $${I}_{ \text{PDraW}}=\int [I(f,{r}_{x}) -{I}_{\text{const}}]$$

Resulting process trends have helped to monitor changes at the early process stages during silica, polyurethane, and zeolite particle formation (data not published). An example for this approach, though calculated differently, is found in [53] in the supplement. During the initial period of an *Escherichia coli* cultivation, scattering is too low to apply PDW theory for data analysis. During this period of low scattering, relative changes to the raw intensity data help to monitor biomass development. As soon as significant scattering occurs, PDW theory can be applied for data analysis. In this regime of multiple light scattering, the raw intensity data is of no further use due to nonlinearity.

## Particle sizing with photon density wave spectroscopy

A challenge for many light scattering analytical techniques is the occurrence of multiple scattering [61]. Traditionally employed techniques such as dynamic light scattering (DLS) and static light scattering (SLS) require highly dilute dispersions for analysis of particle sizes. In contrast, techniques such as time-domain diffusing wave spectroscopy (TD-DWS) [31, 62] and, in particular, PDW spectroscopy are based on multiple scattering theory [2, 12, 63]. In multiple scattering media, the scattering of a single scatterer is overlaid by the scattering of all other scatterers at different positions. Additionally, in highly concentrated dispersions, light is also scattered from directly neighbouring particles, resulting in interference called dependent scattering [62, 64]. Complexity increases when factors such as scatterer shape, polydispersity, concentration, and charge are introduced, which all influence the group scattering behavior. As a result, successfully evaluating measured scattering data with conventional light scattering techniques becomes difficult, if not impossible. The above-mentioned techniques, PDW spectroscopy and TD-DWS, are able to overcome this issue for many types of particle systems [6, 40, 65, 66] and, with some additional effort, can even accurately describe non-classical systems such as soft matter, crystalline samples, or aggregates of particles [6, 48, 67, 68]. We discuss the case for dilute and monodisperse dispersions first before moving on to higher scatterer concentrations and charged systems, where dependent scattering occurs.

### Monodisperse and dilute systems

The reduced scattering coefficient *μ*_s_′ relates to the particle size in the dispersed phase. For a monodisperse and dilute dispersion, Eq. 2 relates the measured reduced scattering coefficient *μ*_s_′ to a particle diameter *d*:

2 $${\mu }_{s}{^{\prime}}=\frac{3 {Q}_{\text{sca}}}{2d}\phi (1-g)$$

**Fig. 4:**
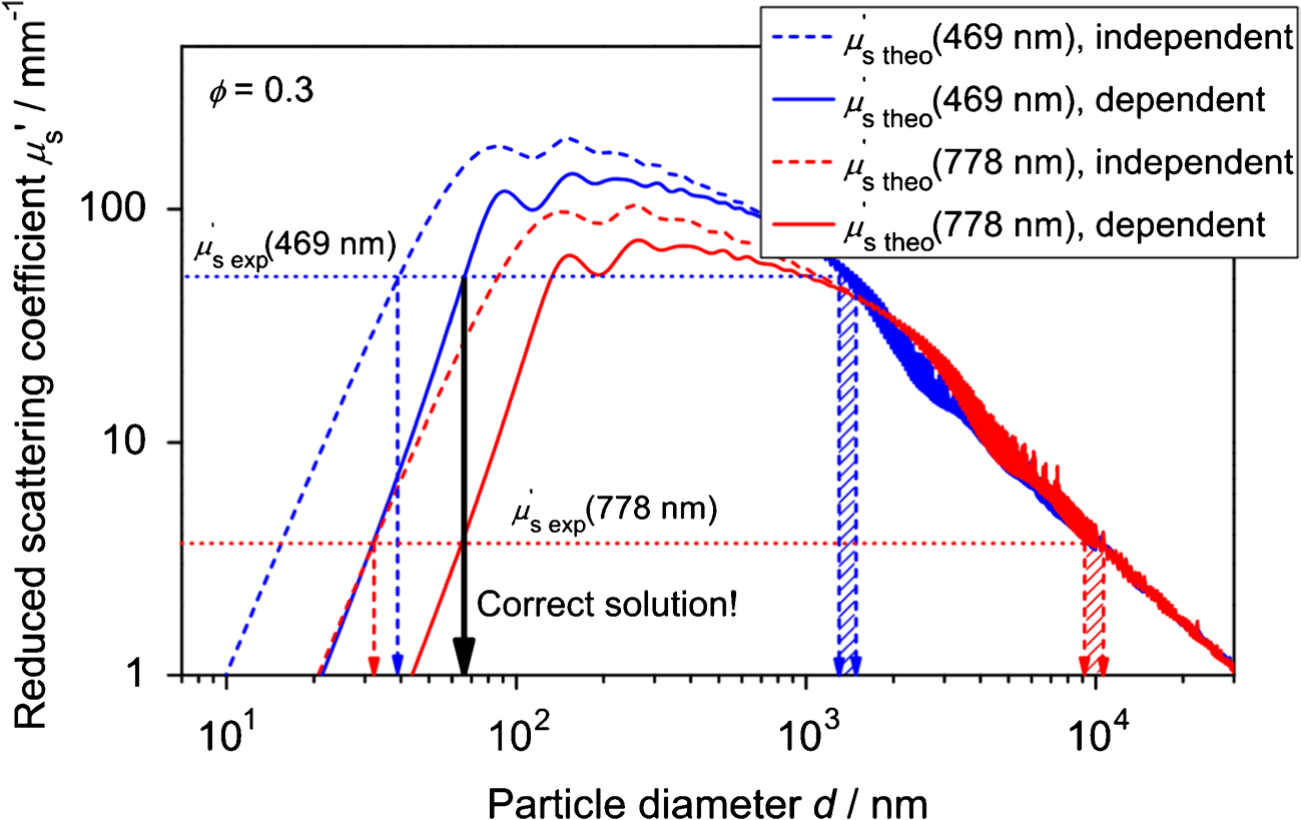
Principle of particle sizing by measurement of *μ*_s_′ with PDW spectroscopy and comparison with theoretical values, calculated by Mie theory in the case of independent light scattering (dashed lines) and for dependent light scattering with the hard-sphere model in the Percus-Yevick approximation (solid lines). Arrows indicate possible size solutions for experimental *μ*_s_′ values. Adapted from [7]

*Q*_sca_ describes the scattering efficiency, and *ϕ* is the volume fraction of the dispersed phase. The parameter *g* is the anisotropy coefficient of scattered light. *Q*_sca_ and *g* are obtained by light scattering theory. For particle sizing by PDW spectroscopy, these parameters are calculated by Mie theory [32, 69]. Other theories, like Rayleigh and Rayleigh-Debye-Gans scattering, are less accurate than Mie theory for particle sizes encountered in turbid dispersions and are, as such, not used. Based on Eq. 2, the theoretical reduced scattering coefficient *μ*′ can be calculated as a function of particle size (Fig. 4). In general, extracting particle sizes from the reduced scattering coefficient of a dispersion also requires the refractive index of the medium *n*_m_ and of the particles *n*_p_. However, *μ*_s_′(*d*) is not a monotonic function; thus, multiple solutions for size exist for an experimentally determined reduced scattering coefficient. If an approximate particle size regime is known (i.e., nanometer or micrometer range), this ambiguity can be resolved. For the size determination by PDW spectroscopy without a priori knowledge, multiple wavelength experiments are required [55], leading to a distinct solution (Fig. 4).

### Dependent scattering and charged systems

**Fig. 5:**
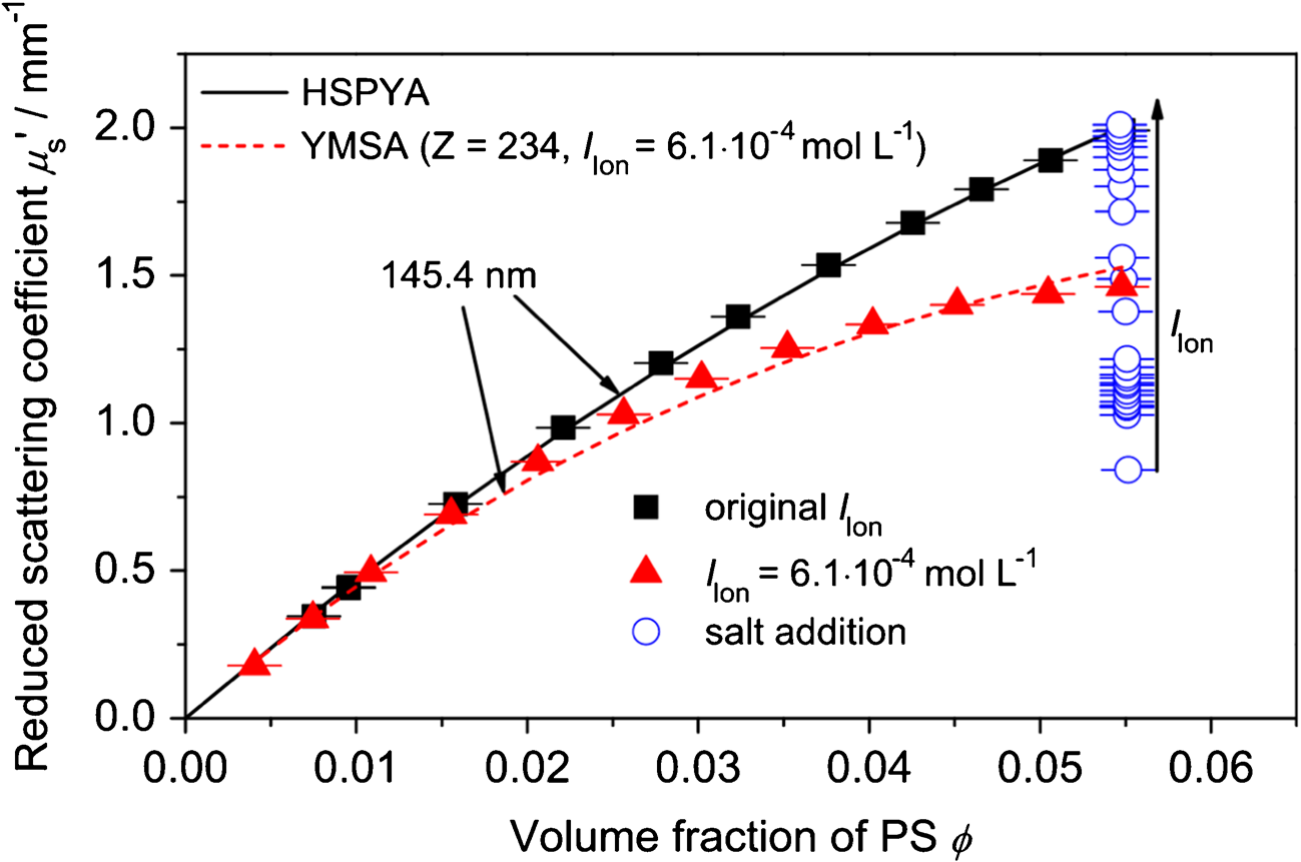
Experimental *μ*_s_′ measured at a wavelength of 979 nm for a polystyrene dispersion with *d* = 145 nm (symbols) in dependence on volume fraction and additionally on NaCl concentration at a volume fraction of 0.055 (open circles). Lines indicate theoretical values from the hard-sphere model in the Percus-Yevick approximation (HSPYA) (solid) and the Yukawa model in the mean sphere approximation (YMSA) (dashed) for *d* = 145.4 nm. Adapted from [7]

As seen in Fig. 5, at high scatterer concentrations, the observed *μ*_s_′ values deviate from linearity. Experimentally measured *μ*_s_′ values do not correlate linearly with scatterer concentration beyond approx. 5 vol% of the scatterer. This phenomenon, known as dependent light scattering [70, 71], arises due to the spatial correlation of particles in the dispersed phase, leading to interference effects in the scattered wave patterns. This interference leads to a relative reduction of the reduced scattering coefficient at high scatterer concentrations. This nonlinear behavior is illustrated in Fig. 5 both in dependency on the volume fraction and ionic strength of a polymer latex dispersion. Without consideration of dependent light scattering, deviations of determined particle size would occur (cf. Fig. 4). This is especially important in industrial processes, where scatterer fractions beyond 5 vol% are commonly encountered.

**Fig. 6:**
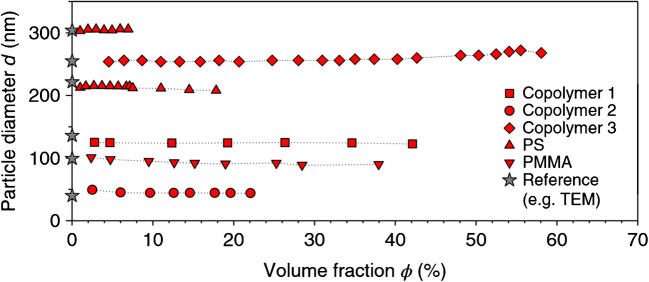
Mean particle size of different aqueous homo- and copolymer particle dispersions at fractions up to 60 vol% measured by PDW spectroscopy and in comparison to reference analysis. Adapted from [9]

One commonly employed model for dependent light scattering is the interference approximation (IA) [64], which can be directly applied to PDW spectroscopy theory. However, selecting an appropriate static structure factor is required, which introduces significant complexity. Equations describing structure factors are called the Ornstein-Zernike equations, and their solutions are henceforth used with the IA in PDW spectroscopy. These analytical solutions are easy to compute and thus well suited to application in rapid data evaluation. One such solution is the hard-sphere model in the Percus-Yevick approximation (HSPYA), which was found to be appropriate for a wide range of particle systems [72]. Bressel [73] expanded the solution by Vrij [74] for polydisperse systems of arbitrary particle size distributions. Limitations exist to this day, and dispersions above the limit of 45 vol% [75] slightly deviate from the HSPYA. With the incorporation of dependent light scattering, the particle size was correctly measured for a wide range of polymer systems, as evidenced in Fig. 6.

When scatterers in media with weak ionic strength have a surface charge, in addition to the effect observed with high volume fractions of particles, the static structure factor experiences further changes which must be accounted for. Contrasting with the HSPYA, the structural factor is additionally affected by the number of charges on the particle *Z*, the inverse debye length *κ*_D_, as well as the relative permittivity of the medium *ϵ*_m_. The Yukawa model in the mean sphere approximation (YMSA) includes all these factors [76, 77]. With an increase in ionic strength, Bressel et al. [7] found that the static structure factor approached the HSPYA model; i.e., the charges were fully screened (Fig. 5).

Application of the YMSA in conjunction with PDW spectroscopy allows for the optical characterization of per-particle charge. Information about the charge state of particle systems is highly industrially relevant. For example, coagulation may be induced by a change in the ionic strength [40, 78].

### Polydisperse systems

As real dispersions are never truly monodisperse, the theoretical reduced scattering coefficient is instead calculated by the sum of scattering by a distribution of particle sizes. An approach to estimating the true distribution is the chi-squared test statistic. Theoretical particle size distributions are used to calculate a theoretical scattering coefficient, which is then matched to experimental values by minimizing *χ*^2^, calculated by Eq. 3:

3 $${\chi }^{2}={\sum }_{\lambda }^{\infty }({\mu }_{\text{s}}{^{\prime}}_{\text{exp},\;\uplambda }-{\mu }_{\text{s}}{^{\prime}}_{\text{theo},\;\uplambda }{)}^{2}/ {\upmu }_{\text{s}}{{{^\prime}}}_{\text{theo},\;\uplambda } \to \text{min}$$

**Fig. 7:**
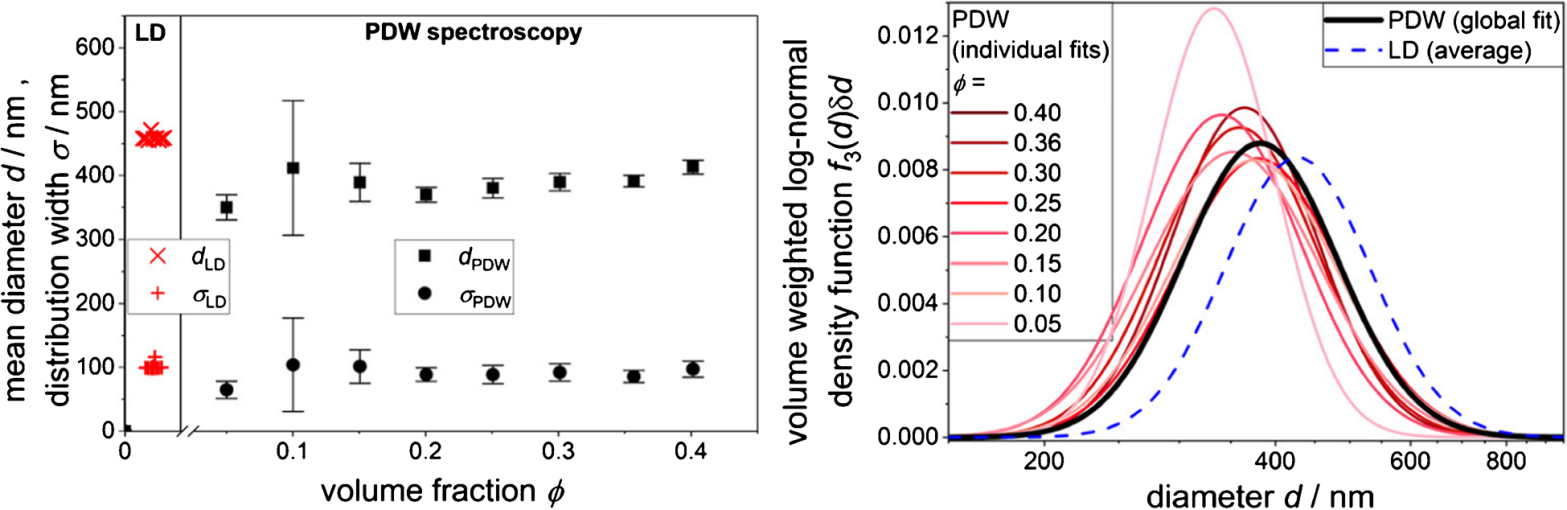
Left: Mean diameter (squares) and distribution width (circles) of an oil-in-water emulsion determined from PDW spectroscopy in dependence on the volume fraction *ϕ* and from dilution-based laser diffraction (LD) (crosses) (by sampling from each PDW spectroscopy dilution step), both based on a volume-weighted log-normal distribution. Errors for PDW spectroscopy data are derived from individual fits to *μ*_s_′(λ). Right: Associated distribution functions from the left figure for LD (dashed line) and from PDW spectroscopy (individual fit, thin lines; global fit, thick line). Adapted from [46]

Specifically, the minimization is applied to all measured wavelengths utilizing a Levenberg-Marquardt algorithm. Common distribution functions, like Gaussian or log-normal, require only two parameters. Measuring *μ*_s_′ with multiple wavelengths aids in fitting these two parameters. Different distribution types were evaluated and were found to have a significant effect on calculated *χ*^2^. The distribution which best fit the underlying particle size distribution (PSD) has the lowest *χ*^2^ values [55]. Levenberg-Marquardt algorithms may lead to erroneous solutions if the initial parameters are poorly chosen. Convergence is reliably achieved for initial parameters near the expected size distribution values. Good agreements with reference analytics, e.g., laser diffraction, electron microscopy, and other analytical techniques, were found [38, 46]. An example of successful distribution measurement for fractions from 5 to 40 vol% is displayed in Fig. 7 for an oil-in-water emulsion.

### Bidisperse, nonspherical, or aggregated systems

The following methods are non-standard and have been applied only to specific systems, and yet their listing and explanation should serve as an inspiration for future applications and development of PDW spectroscopy. Their main problems are related to the number of unknown parameters and measurement error interfering with data evaluation. First, systems containing two populations of two particle sizes, i.e., bidisperse systems, will be explored.

When two populations of narrowly distributed particles are present, the static structure factor and, as a result, dependent light scattering are highly affected. Such systems are, for example, high-volume fraction, low-viscosity polymer latices. Fitting data is analogous to polydisperse systems, i.e., through *χ*^2^ minimization, but more parameters are used. Mainly, the interest lies in the relative number density of particles and their respective (monodisperse) particle sizes. Modelling is not trivial, but analogous to the determination of the structure factor for polydisperse systems [22]. A significant limitation of this method exists; the scaling of dependent scattering in dependence on the number density of large particles is almost logarithmic. Thus, differences between relative number densities of large particles from 0.5 to 1 have a minor effect on the dependent scattering, on the order of measurement error. As a result, determining the particle size of small particles is difficult if a large volume fraction of larger sized particles is also present [7, 22]. A deconvolution algorithm implemented by Eliçabe et al. [79] for turbidimetric measurements could be used in the future to expand the measurement range of relative number densities. Future work should additionally focus on reducing measurement and analysis error.

Previously discussed models for particle sizing assumed spherical particles. There are still two gaps to fill: aggregates of particles and nonspherical particles. The latter is of interest for crystallization experiments, but no model has been implemented yet [49]. In the case where particle geometry is known, the work by Mischenko on the T-matrix method offers remarkably accurate results for relevant scattering parameters [80]. Due to the nature of particle dispersions, random orientation of particles occurs, to which solutions exist and may be employed. It must, however, be stated that this approach is computationally expensive compared to the Mie solution of spherical scatterers. New, computationally fast approximations of the static structure factor are required, and such models are currently being developed [72, 81, 82]. A major drawback, however, is the lack of solutions to polymodal or polydisperse dispersions for particles of arbitrary geometry, requiring further research [83]. Currently, an equivalent spherical diameter is calculated, i.e., spheres of size *d* exhibiting identical scattering properties to the measured dispersion containing nonspherical particles.

More interestingly, a recent development has allowed the calculation of the radius of gyration *a*_G_ and fractal dimension *D*_f_ of fractal aggregates consisting of primary particles. Such fractal aggregates are, for example, pyrogenic or precipitated metal oxides [66, 68, 84]. All of the above systems have well-behaved fractality *D*_f_ of the order 1.5 < *D*_f_ < 3. Light scattering of primary particles (< 20 nm) in an aggregate is well approximated by Rayleigh-Debye-Gans theory with minimal deviation from Mie theory. Solutions to such problems are known in literature, but were previously limited to *D*_f_ < 2 [85]. Zimmerman et al. expanded previous solutions to include aggregates of higher fractal dimensions and also calculated the anisotropy coefficient, $g$, which then allowed for size interpretation with PDW spectroscopy. Process measurements, however, were not yet explored [68].

## Current technical status of PDW spectroscopy for PAT applications

PDW spectrometers are commercially available from PDW Analytics GmbH, Potsdam, Germany. For scientific collaborations, they are also currently accessible at the University of Potsdam, Germany; the Technical University of Berlin, Germany; and the Zurich University of Applied Sciences, Switzerland. A state-of-the-art commercial PDW spectrometer provides one to four operating laser wavelengths in the typical range of 600 to 1000 nm. The experimental wavelengths are selected based on the best suitability for monitoring the process of interest. For example, for materials exhibiting very low turbidities, e.g., during silica synthesis, spectrometers have been equipped with lasers in the 400 nm range. The latest technological advancements during the recent years are mainly the instrument’s compactness, the solution for a fiber optical plug for PDW spectroscopy, and the virtualization as well as OPC capability of the control software.

**Fig. 8:**
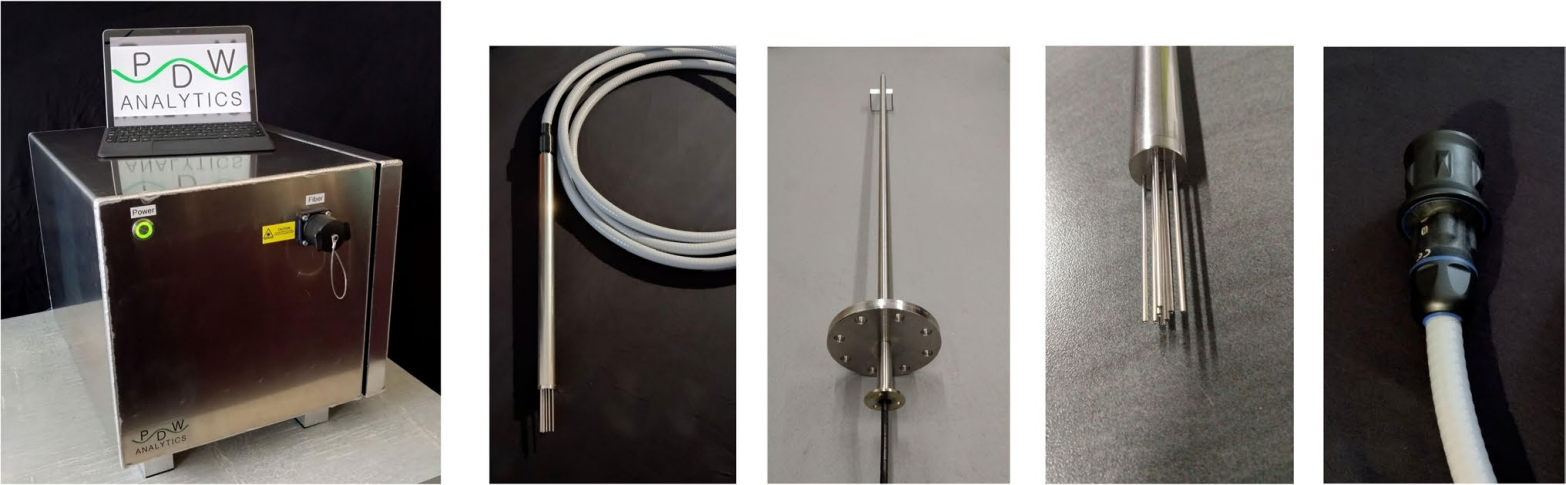
Left: Front view of a state-of-the-art PDW spectrometer with power button and fiber-optical plug for the process probe connection with control tablet on top. Second left to right: Standard 25 × 450 mm PDW process probe with fiber optical conduit, 25 × 1600 mm PDW process probe with DN80 flange, probe tip of a 19 × 450 mm PDW process probe, and process probe plug for spectrometer connection

The current dimensions of a PDW spectrometer are 415 × 500 × 395 mm (*w* × *d* × *h*), weighing approx. 19 kg (Fig. 8). The instrument is rated at IP54. A PDW spectrometer is operated at 24 VDC, with 30 W electrical power needed (by an external short-circuit-protected safety isolating transformer for 230 VAC sources or direct DC supply). The instrument is equipped with a USB or LAN port and a fiber-optical port for the process probe connection. The instrument can either be operated directly with a control computer or by a virtual machine.

The laser light from the spectrometer is guided through optical fibers to the material or the process of interest. The optical fibers also act as light collectors, thus guiding light back from the material or the process into the spectrometer. For a successful PDW spectroscopy experiment, a spatial variation of distances between the light emission point inside the material and the light detection point is required. Two technical realization concepts currently exist for this demand: (a) a variation of the distance of one emission and one detection fiber or (b) a multitude of spatially fixed fibers, providing a distance variation. For process monitoring, concept (b) is used currently, since it avoids any moving or electronic parts and is a purely fiber optical probe concept. Except for the optical fibers, no further optical components like focusing elements and optical filters are required, making the process probes relatively inexpensive compared to other advanced optical PAT probes. Due to the use of only fiber optics, process probes can be easily scaled in length and equipped with any flange or used with compression fittings. For example, the longest process probe manufactured so far has been a 1.6-m probe equipped with a DN80 flange for reactor implementation at cubic meter scale. However, the standard probe length is 45 cm. The length of the fiber optical conduit from the probe ranges typically from 5 to 10 m. The maximum length for the fiber optical conduit without loss of data quality still requires evaluation. The typical distances between emission and detection points scale from 3 to 20 mm. Thus, PDW process probes require a certain minimal diameter. Currently available probe designs are either 19 or 25 mm in diameter (Fig. 8); standard material currently is 1.4301 stainless steel.

The PDW process probes are not yet rated with respect to temperature, pressure, and pH conditions. Thus, they should only be implemented in processes after a risk assessment, ideally in R&D and pilot scale environments. Based on experience, the probes can be operated from −25 to 100 °C, for a short time up to 125 °C, up to 3 bar, and between pH 0 and 14. While each single physical condition poses no risk for the PDW process probe, the combination of environmental factors can have an impact. For example, during very harsh synthesis conditions for zeolite L (160 °C, pH 14, 6 bar, 42 h batch time) [49] the glass of the optical fibers slowly dissolved; thus, only a few batches could be monitored by a single PDW process probe. Either a “single use” concept or chemically resistant optical fibers may overcome the chemical limitation of glass. It has also been observed that sterilization-in-place in biotechnological applications limits the lifetime of PDW process probes, though cycle times cannot be stated yet. Defined and improved process probe specifications are one of the main tasks for the further development of the PDW technology for PAT.

The instrument control software is currently Windows-based, includes data generation and data analysis, and is designed for continuous measurement and data analysis. Batch-specific raw data is immediately and permanently stored so that data loss cannot occur. Raw data analysis towards the optical coefficients and particle size is executed in real time. It can also be analyzed post-process without changes to the raw data. The data is stored in a non-proprietary file format so that the files can be read and processed by any further software. The software supports OPC for permanent data export. Relevant process parameters are the absorption and the reduced scattering coefficients per wavelength as well as the particle size and the distribution width, all provided as a function of time in real-time, i.e., a time resolution of approx. 1 min per set of optical coefficients. The time resolution can be adjusted (approx. 15 s to 4 min) depending on the number of lasers used as well as the number of fiber distances and modulation frequencies applied. Faster measurements mainly reduce the precision of the optical coefficients. In practice, time resolution is adjusted in that range to the temporal dynamics within the process of interest.

Regarding explosive environments, the spectrometer itself is currently not ATEX rated. Implementation in an explosive zone would therefore require either special development or other protective measures. However, as the PDW process probe technology has no electrical components, it is intrinsically ATEX safe. Regarding optical ATEX safety, the optical emission from the spectrometer and probe is below 5 mW, without any further focusing optics. Thus, a zone separation between the PDW spectrometer and the PDW process probe can be considered. Regarding laser safety, even though the emission ex instrument is below 5 mW, the lasers should only be operated when the PDW process probe is implemented into the reaction vessel and only for actual material or process characterization. The lasers themselves only emit light after an intentional start of a measurement.

For implementation and maintenance, IQ/OQ/PQ procedures are in place. The spectrometer should be serviced yearly by the manufacturer, mainly evaluating laser emission quality in the individual fiber optical channels. The PDW process probes do not need to undergo regular service intervals apart from optical checks for visible damage. In case of obvious signs of wear, e.g., bent fiber tips, the fiber distances need to be requalified. The main harm to the process probe from physical impact can be the delocalization of the positions of the optical fibers and their mechanical damage.

The costs for PDW spectrometers differ depending on the exact specifications, but the costs are found in the same range as established optical PAT solutions such as process microscopes, NIR, or RAMAN spectrometers and similar advanced PAT. However, the cost for a PDW process probe is typically lower. This opens the attractive paths towards probe multiplexing, i.e., one spectrometer operating several process probes. Based on the current spectrometer concept, this is currently only feasible in a serial way, i.e., the read-out of one probe after the other and therefore decreasing the time resolution per process probe. While this probe multiplexing is technically feasible, it will only be realized demand-driven.

## Applications of PDW spectroscopy as PAT

PDW spectroscopy has been applied as PAT in various industries with a focus on inline measurements in recent years. A comprehensive overview of the scientifically published applications is shown in Table 1.

**Table 1:** Overview of scientifically published PAT applications of PDW spectroscopy

System	Application*	Reactor scale	Particle size**	Max. particle concentration**	Reference methods***	Reference
Chemistry						
Polymerization	Solvent-borne acrylic copolymer formation	1 L	n.r.	50 wt%	Torque measurements	[35]
	Emulsion copolymerization of vinyl acetate/Versa® 10	1.2 L	70–416 nm	63 wt%	CHDF, DLS, sedimentation	[36]
		1–100 L	51–326 nm	67 wt%	DLS, Raman, sedimentation	[39]
		1–100 L	n.r.	62 vol%	DLS	[43]
	Emulsion polymerization of vinyl acetate	1 L	50–600 nm	54 wt%	DLS, SLS	[37]
		1 L	271–412 nm	55 wt%	DLS, SLS	[38]
	Seeded emulsion polymerization of styrene	1 L	49–296 nm	37 wt%	DLS, SLS	[38]
	Seeded emulsion copolymerization of MMA/BA/MAA	1 L	45–425 nm	40 wt%	CHDF, DLS, RI	[40]
		1 L	30–225 nm	45 wt%	DLS, TUS	[41]
		1 L	30–300 nm	45 wt%	DLS, RI	[42]
	Suspension copolymerization of styrene and divinylbenzene	400 L	6–14 µm	41 vol%	None	[55]
	Starved feed copolymerization of styrene and acrylic monomers	25 L	50–250 nm	42 vol%	DLS	[34]
	Formazine synthesis	1.2 L	n.r.	n.r.	Turbidity	[60]
Precipitation	Titania synthesis	1 L	240 nm	n.r.	DLS, SEM, gravimetry	[66]
	Titania disintegration	1 L	169–560 nm	n.r.	DLS, SEM, gravimetry	[84]
	Barium sulfate precipitation	1 L	n.r.	n.r.	None	[9]
Crystallization	Zeolite A and L synthesis	1.5 L	n.r.	n.r.	SEM, XRD	[49]
Others	Poly(*N*-isopropylacrylamide) coil-to-globule transition	1 L	100–200 µm	2.4 wt%	FBRM, PVM, turbidity	[67]
		1 L	n.r.	n.r.	None	[56]
Food technology						
Emulsification	Oil-in-water emulsion depletion flocculation	1 L	400 nm	40 vol%	LD, rheology, UV/VIS	[46]
	Oil-in-water emulsification	1 L	60–400 µm	35 vol%	None	[44]
Crystallization	Lactose crystallization	1 L	60–140 µm	60 wt%	FBRM, PVM	[48]
	Milk fat phase transition	1 L	n.r.	3.5 wt%	DSC	[47]
Enzymatic conversion	Milk coagulation	1 L	n.r.	3.5 wt%	None	[54]
	Beer mashing	5 L	n.r.	n.r.	None	[54]
Biotechnology						
Fermentation	*Escherichia coli* fermentation	3.7 L	0.5–2 µm	69 g L^−1^ CDW	Gravimetry, OD, TCD	[53]
	*Ralstonia eutropha* fermentation	6.6 L	0.5–2 µm	75 g L^−1^ CDW	Gravimetry, HPLC, GC	[50, 52]
Algae cultivation	*Scenedesmus rubescens* cultivation	4.8 m^2^	1–12 µm	33 g L^−1^ CDW	Gravimetry, cell volume	[51]
Cosmetics						
Emulsification	Phase inversion temperature emulsifications	1 L	20–30 µm and 120–240 nm	20 vol%	DLS, light microscope	[45, 55]

*BA butyl acrylate, *MAA* methacrylic acid, *MMA* methyl methacrylate

**n.r. not reported, *CDW* cell dry weight

***CHDF capillary hydrodynamic fractionation, *DLS* dynamic light scattering, *DSC* differential scanning calorimetry, *FBRM* focused beam reflectance measurements, *GC* gas chromatography, *HPLC* high-performance liquid chromatography, *LD* laser diffraction, *OD* optical density, *PVM* particle vision microscopy, *RI* refractive index, *SEM* scanning electron microscopy, *SLS* static light scattering, *TCD* total cell density, *TUS* turbidity spectroscopy, *UV/Vis* ultraviolet and visible light, *XRD* X-ray diffraction

### Chemistry — polymerization

Hass et al. investigated the industrial scale suspension polymerization (400 L) of a styrene-divinylbenzene copolymer with a volume fraction of above 40 vol% [55]. The initial process observed was homogenization of the monomer emulsion before polymerization, where a decrease in droplet size was observed with PDW spectroscopy. They identified that homogenization was not complete even after 16 h of stirring, which was thought to be the case in the standard synthesis protocol. In a follow-up synthesis, equilibrium of droplet size was achieved by monitoring *μ*_s_′ until it was constant; then, synthesis was started by thermal degradation of the radical initiator. Further model development is needed, as the particle size strongly depends on the refractive index of the scatterer, which changed over time due to the polymerization process.

The application of PDW spectroscopy for inline monitoring of solvent-borne acrylic copolymer dispersion formation via the acetone process was investigated by Kutlug et al. in a 1-L laboratory-scale reactor [35]. PDW spectroscopy also demonstrated sensitivity to variations in temperature, degree of acrylic acid neutralization, and water feeding rate, offering insights into polymer solubility and dispersion stability. Unlike conventional methods requiring sample removal and dilution, PDW spectroscopy enabled continuous, undisturbed monitoring of the process of water addition. The findings in this work indicate that torque and viscosity are both insufficient indicators of phase inversion, since it was found that light scattering structures form and vanish at lower added volumes of water than the observed change in relative torque.

**Fig. 9:**
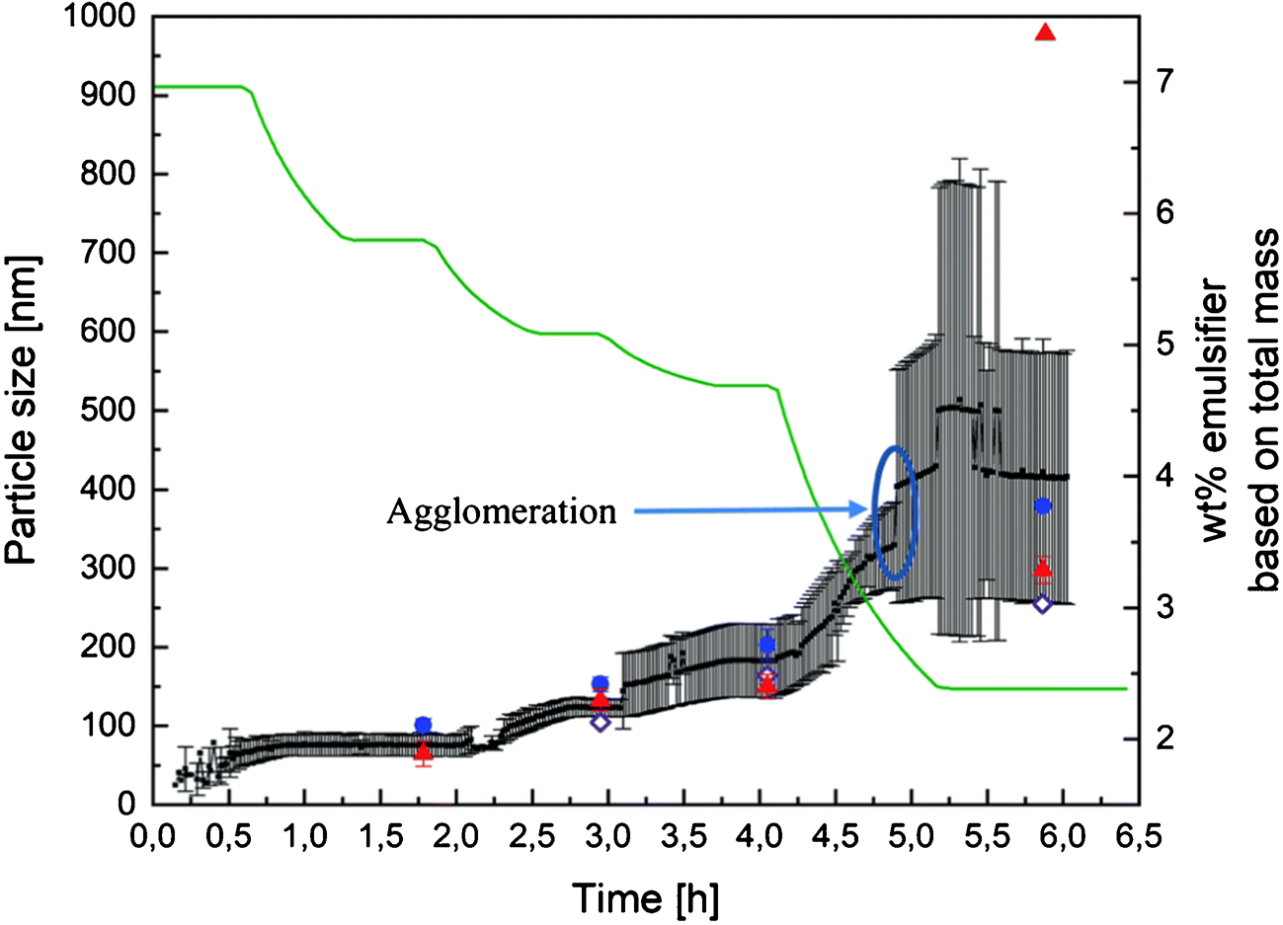
Progression of particle sizes measured via PDW spectroscopy (black), DLS (blue), sedimentation analysis (red), and calculated theoretical particle size (purple) as well as emulsifier content (green) during the four-step emulsion polymerization. From [36]

**Fig. 10:**
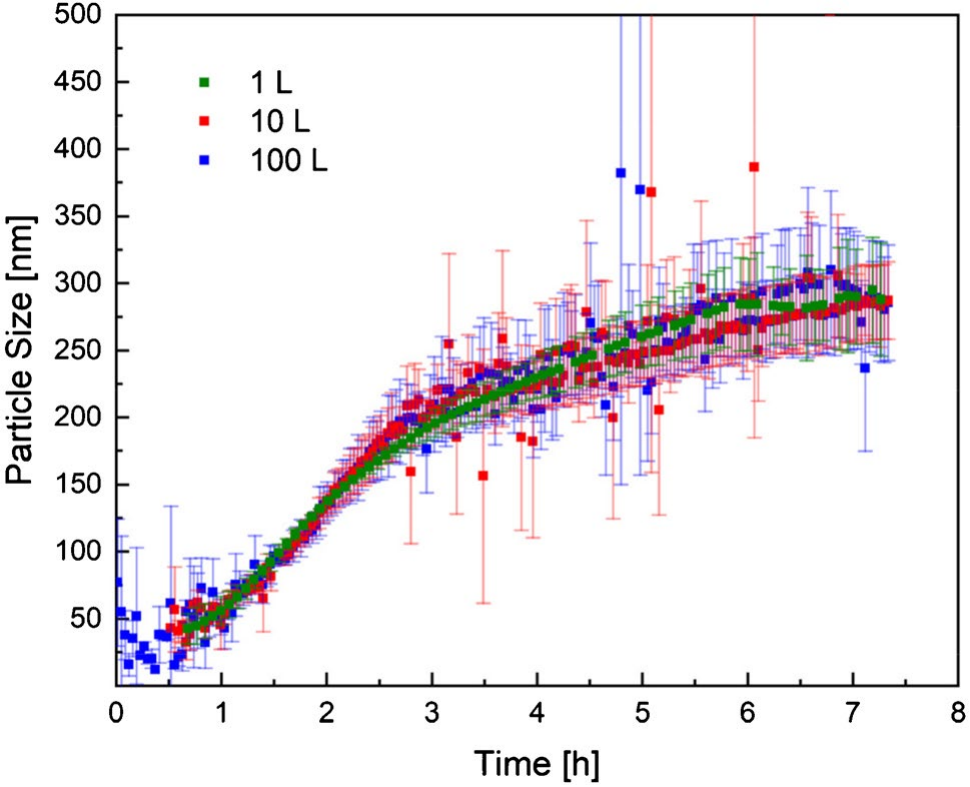
Development of particle size over reaction time in 1 L (green), 10 L (red), and 100 L (blue) reactor scales, measured inline via PDW spectroscopy. From [39]

Jacob et al. [36, 39] demonstrated a successful and reproducible scale-up of VAc and Versa®10 emulsion copolymerization from 1 to 100 L, achieving polymer contents of up to 67 wt%. PDW spectroscopy was demonstrated to deliver accurate and reproducible results at high solid contents, continuing to provide reliable measurements even beyond 36 wt% polymer content, where other reference techniques (DLS and disc centrifuge sedimentation analysis) struggled due to agglomeration and multiple light scattering (Fig. 9) [36]. It was stated that severe probe fouling did not impact the measurement quality. In the experiments of scale variation of 1 L, 10 L, and 100 L, a nearly similar growth of particle size was achieved for each scale (Fig. 10) [39].

Schlappa et al. investigated the application of PDW spectroscopy for inline monitoring of VAc emulsion polymerization at high solid contents (> 40 wt%) in a 1-L laboratory reactor [37]. The study identified three key polymerization stages based on PDW spectroscopy data: rapid particle formation, slower particle growth, and stabilization upon completion of monomer addition. In addition, PDW spectroscopy detected unexpected particle aggregation and gelation, demonstrating its value for process control and ability to detect process deviations. During emulsion polymerization, PDW spectroscopy provided reliable size measurements for PS dispersions but faced challenges in analyzing PVAc particles due to water swelling, which altered their refractive index and density. However, an advanced particle size analysis was developed to account for water swelling, improving particle size determination by up to a factor of ten [38]. In a follow-up study, insights into the dynamics and mixing of VAc/Versa®10 copolymerization were extended in reactors up to 100 L [43]. It was revealed that while polymerization dynamics are comparable up to a polymer volume fraction of approx. 15 vol%, deviations at higher fractions indicate differences in particle growth and mixing efficiency.

Aspiazu et al. evaluated PDW spectroscopy for inline monitoring of particle sizes during seeded semibatch emulsion copolymerization of MMA, BA, and MAA in a 1-L laboratory reactor [40]. They demonstrated that PDW spectroscopy effectively tracks particle growth and detects process deviations, such as particle aggregation and secondary nucleation. The study found that PDW spectroscopy performs well for a broad range of particle sizes and solid contents but struggles with sizes above 350 nm and solid contents exceeding 40 wt% due to multiple size solution ambiguities and very high light scattering. The inline sensitivity of PDW spectroscopy for detecting bimodal particle distributions is limited, but it successfully identifies secondary nucleation through scattering variations at lower wavelengths. Asua [11] critically summarized that while PDW spectroscopy provides accurate mean size data under controlled conditions, its inability to track particle size distributions under evolving physico-chemical system properties constrains its utility for comprehensive online control strategies in industrial emulsion polymerizations. For example, in seeded semibatch emulsion copolymerization, detection of secondary nucleation is found to be challenging while particle coagulation is quickly monitored, allowing preventive actions in industrial polymerization processes [40]. In a follow-up study, the authors examined how measurement parameters (fiber distances and laser modulation frequencies) and uncertainties in sample properties (refractive index, density, and solids content) affect the accuracy of particle size determination [42]. Particle size measurements were found to be more sensitive to uncertainties in refractive index and solid content than to density variations. The findings suggest that optimizing measurement distances and modulation frequencies, and accounting for real-time changes in solids content and refractive index, significantly enhances the accuracy of particle sizing by PDW spectroscopy for monitoring polymerization processes.

Münzberg et al. investigated the synthesis of formazine as an ISO standard calibration material for turbidity measurements. The syntheses were executed in a 1.2-L laboratory reactor [60]. Though stirring is not required by the ISO norm, it was shown that synthesis without stirring causes changes to this calibration standard. Reaction temperature variation indicated a strong influence of temperature on the onset time of formazine precipitation, resulting in an optically determined activation energy. Despite this effect on the onset of precipitation, no influence of temperature on the turbidity was found after 24 h. A systematic variation of precursor concentrations revealed a non-linear effect on the precipitation onset and a linear dependency for the scattering after 24 h. Although an industry standard for calibration, formazine suspensions provide a *μ*_s_′ of approx. 0.2 mm⁻^1^, depending on measured wavelengths, for the ISO standard concentration and a *μ*_s_′ of approx. 0.6 mm⁻^1^, from the maximum soluble concentration of precursors. Because many real-world chemical and biotechnological processes involve much higher turbidities, formazine has been found unsuitable for calibrating instruments intended for such conditions.

### Chemistry — crystallization and precipitation

Crystallization and precipitation, as essential process steps in the chemical industry, have also been monitored with PDW spectroscopy. In particular, inline PDW spectroscopy was applied to zeolite synthesis [49], titania synthesis [66], titania disintegration [84], and barium sulfate precipitation [9].

**Fig. 11:**
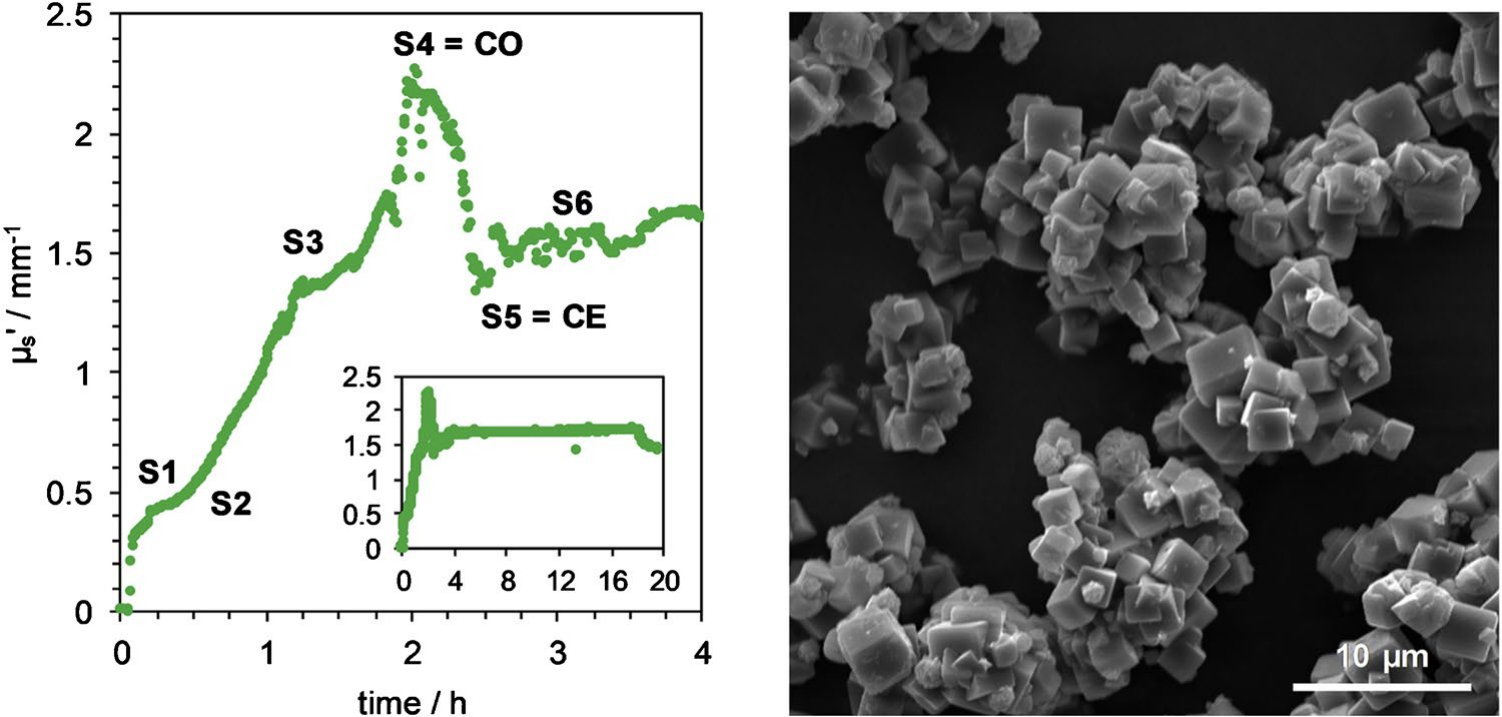
Left: Reduced scattering coefficient at 636 nm during the first 4 h of zeolite A synthesis, with sampling points S1–S6 marked; sample S4 corresponds to the crystallization onset (CO), and S5 to the crystallization end (CE). The inlet displays the complete standard reaction time of 20 h. Right: The electron microscope image shows the final zeolite A product after 20 h of synthesis. From [49]

The paper by Häne et al. explores the use of PDW spectroscopy for real-time inline monitoring of zeolite A and zeolite L synthesis in a 1.5-L stainless steel reactor [49]. The crystallization process was successfully tracked by measuring the reduced scattering coefficient, revealing distinct stages from amorphous particle formation to crystalline zeolite structures. The study demonstrated that the onset and completion of crystallization could be tracked with PDW spectroscopy, correlating well with offline analyses like X-ray diffraction (XRD) and scanning electron microscopy (SEM). An early end of crystallization after approx. 4 h could be detected using PDW spectroscopy, whereby the standard synthesis protocol required a synthesis time of 20 h (Fig. 11). The influence of molar water content was also examined, showing a linear increase in crystallization time with higher water content, while different silica sources led to significant variations in crystal formation.

Titania (TiO₂) particle synthesis via the hydrolysis of tetraethyl orthotitanate in the presence of potassium chloride was monitored by inline PDW spectroscopy in a 1-L laboratory reactor [66]. The measurements revealed an unexpected disintegration of titania secondary particles over time, leading to a decrease in particle yield. The addition of hexadecylamine was found to prevent this disintegration, increasing particle yield by 44% after 24 h. DLS and zeta-potential measurements confirmed that hexadecylamine reduces the electrostatic repulsion between primary particles, stabilizing the secondary aggregates. SEM images indicated that the titania particles were porous aggregates of smaller primary particles, supporting a growth-by-aggregation mechanism.

The effect of alkali metal salt addition on the disintegration of titania secondary particles into their primary particles during precipitation from tetraethyl orthotitanate in ethanol was examined by Zimmermann et al. [84]. PDW spectroscopy was used inline to monitor particle size changes, revealing that higher concentrations of alkali metal salts, particularly potassium chloride and cesium chloride, accelerate disintegration by increasing electrostatic repulsion among primary particles. The study found that larger cations, such as cesium ions (Cs⁺), promote faster disintegration compared to sodium ions (Na⁺), while sulfate ions (SO₄^2^⁻) prevent disintegration by reducing the particle charge. SEM and DLS confirmed that higher alkali chloride concentrations lead to smaller secondary particles and lower yields, with hydrochloric acid addition completely breaking down aggregates into transparent colloidal dispersions of 4.7 nm primary particles. The findings suggest that salt concentration and type can be used to control the stability and size distribution of titania particles in sol-gel processes [84].

In another study, an optical model to enable in situ characterization of fractal aggregates using PDW spectroscopy was developed by Zimmermann et al. [68]. Measurements were conducted on aggregated amorphous silica and titania nanoparticles in different solvents, and the results were validated against DLS measurements. The model successfully linked the reduced scattering coefficient to key fractal properties, including the radius of gyration and fractal dimension, allowing determination of the hydrodynamic radius of aggregates. While PDW spectroscopy and DLS results showed good agreement for most samples, deviations were observed for titania aggregates with high primary particle interactions, suggesting limitations in the applicability. The study highlights PDW spectroscopy as a promising tool for real-time aggregate characterization, with potential improvements in accounting for polydispersity and dependent scattering effects.

Fifty precipitation experiments of barium sulfate were monitored on the basis of a design of experiments (DoE) approach in a 1-L lab reactor scale. The influence of sulfate and barium ions on the particle formation was monitored with PDW spectroscopy [9]. Hass et al. found that the barium sulfate suspensions undergo significant changes for several hours even after the initially rapid precipitation reaction. The absorption coefficient *μ*_a_ remained constant during synthesis, while the reduced scattering coefficient *μ*_s_′ evolved over time. The specific reduced scattering coefficient *τ*, which is the quotient of *μ*_s_′ and the volume fraction, was used to identify the variation in particle formation regardless of differing volume fractions. Particle size was not investigated, but process deviations were clearly visible and significant.

### Chemistry — others

The coil-to-globule transition of poly(*N*-isopropylacrylamide) microgel particles in concentrated aqueous suspensions was monitored by inline PDW spectroscopy in a 1-L laboratory reactor at polymer concentrations of up to 2.4 wt% [56, 67]. The studies showed that PDW spectroscopy effectively tracked polymer hydration and dehydration, demonstrating an inverse hysteresis effect, where the lower critical solution temperature shifts occurred at different temperatures depending on heating or cooling rates. Compared to other PATs such as focused beam reflectance measurement (FBRM), process microscopy, and turbidity measurements, PDW spectroscopy provided the most sensitive detection of polymer phase changes.

### Food technology — crystallization, enzymatic conversion, emulsification

Food production involves many different processing steps, which can be optimized by precise process control. PDW spectroscopy has been applied in different fields in food processes: beer mashing [54], oil-in-water emulsification [44, 46], lactose crystallization, enzymatic milk coagulation [54], and milk fat phase transition [47].

Hartwig and Hass [48] investigated the application of PDW spectroscopy for in-line monitoring of lactose crystallization at industrially relevant concentrations (25–60 wt%) in a 1-L reactor. By independently measuring absorption and reduced scattering coefficients, PDW spectroscopy effectively determined solubility and nucleation points, with reproducible trends confirmed across repeated experiments and varying cooling rates (0.1 vs. 2 K min⁻^1^). PDW spectroscopy data revealed clear decreases in scattering during dissolution and captured the widening of the metastable zone at faster cooling, demonstrating sensitivity to crystallization kinetics. Importantly, under severe probe fouling, where FBRM and PVM signals became noisy and inconsistent, PDW spectroscopy measurements remained smooth and reproducible, owing to its extended sensing zone. These experimental results highlight the efficacy of PDW spectroscopy as a robust and accurate PAT for monitoring crystallization in highly concentrated systems, where process sensors are prone to probe fouling.

Vargas Ruiz et al. [47] investigated the use of PDW spectroscopy for inline monitoring of milk fat phase transitions during thermal processing in a 1-L laboratory reactor. The study identified characteristic breakpoints in scattering behavior at 16 °C and 24 °C, corresponding to the crystallization and melting of milk fat, respectively. Real-time, dilution-free insights into structural changes in milk were accessible, distinguishing PDW spectroscopy from conventional methods like offline differential scanning calorimetry (DSC). The technique was applied to low-fat (0.1%) and full-fat (3.5%) milk, confirming that light scattering was strongly influenced by fat globule transitions, whereas fat-free milk showed different scattering trends due to casein micelle interactions.

**Fig. 12:**
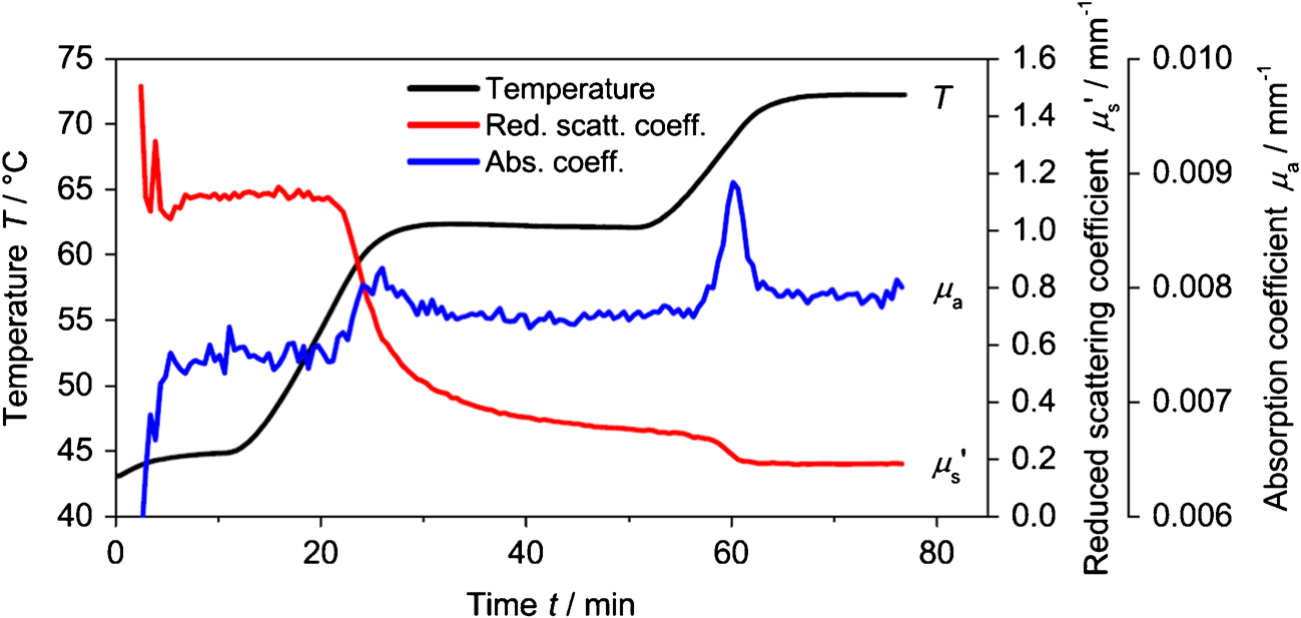
Temperature, reduced scattering coefficient, and absorption coefficient from PDW spectroscopy during the enzymatic conversion of beer mash. From [54]

The study by Hass et al. [51] demonstrates how PDW spectroscopy provides real-time insights into enzyme-induced milk coagulation, with chymosin added to fresh milk containing 0.1–3.5 wt% fat. Changes in scattering properties enabled monitoring of the coagulation process and the effects of enzyme concentration and temperature. PDW spectroscopy was further applied to beer mashing, where swelling and partial dissolution of malt particles were captured through inline changes in the optical coefficients (Fig. 12). Notably, the inline data identified the enzymatic activation temperature at 58 °C, detected below the set rest temperature at 62 °C, and allowed continuous tracking of starch degradation dynamics as a function of time during the mashing rest.

Inline monitoring of the influence of emulsifier concentrations and stirring speeds on the sunflower oil in water emulsification process with PDW spectroscopy was analyzed by Reich et al. [44]. Mean droplet sizes during the emulsification process were obtained by PDW spectroscopy at oil fractions of 35 vol%, and expected droplet size changes induced by varying emulsifier concentrations and stirring speeds could be confirmed in real time.

The paper by Bressel et al. [46] investigates depletion-induced flocculation in concentrated oil-in-water emulsions using PDW spectroscopy in a 1-L laboratory reactor (cf. Fig. 7), at fractions ranging from 5 to 40 vol%. It was applied to measure light scattering and absorption in emulsions stabilized with polysorbate 80, revealing structural changes in real time without the need for dilution. The study explored the effects of xanthan, a semi-flexible polymer, on flocculation, showing that an increasing xanthan concentration led to different aggregation behaviors, including rapid structural formation at 0.2 wt% and gel-like stabilization at higher concentrations. Three flocculation stages were identified: an initial sharp drop in the reduced scattering coefficient, a slower power-law decrease linked to spinodal demixing, and a final aggregation phase. The method also demonstrated sensitivity to changes in temperature, with lower temperatures slowing the depletion process and allowing more detailed time resolution of the flocculation kinetics. These findings establish PDW spectroscopy as a tool for inline monitoring of flocculation and structural evolution in concentrated emulsions.

### Biotechnology — fermentation, algae cultivation

The first results for biomass concentration measurement using offline PDW spectroscopy were shown by Engelhard et al. [86] for a dilution series of a brewer’s yeast suspension. Since then, the application of inline PDW spectroscopy has been evaluated in different biotechnological processes in bioreactor cultivations of the bacteria *Escherichia coli* and *Ralstonia eutropha* as well as the phototrophic microalgae *Scenedesmus rubescens* [50–53].

It was shown that *μ*_s_′ measured with PDW spectroscopy accurately predicts *E. coli* biomass formation in a range of 5–69 g L^−1^ cell dry weight and is as reliable as other inline or atline optical methods such as manually diluted photometric measurements, automated diluted photometric measurements, and inline transmission-based turbidity measurements. In that range, inline measured growth rates of *E. coli* were obtained by PDW spectroscopy, thus allowing for real-time feed control. Additionally, at biomass concentrations below the limit of detection for *μ*_s_′ (approx. 5 g L^−1^), changes in the raw light intensity values provided real-time information of biomass formation. By associating *μ*_s_′ with *μ*_a_, inline, time-independent detection of process deviations from the “golden batch” trend becomes available with PDW spectroscopy [53].

**Fig. 13:**
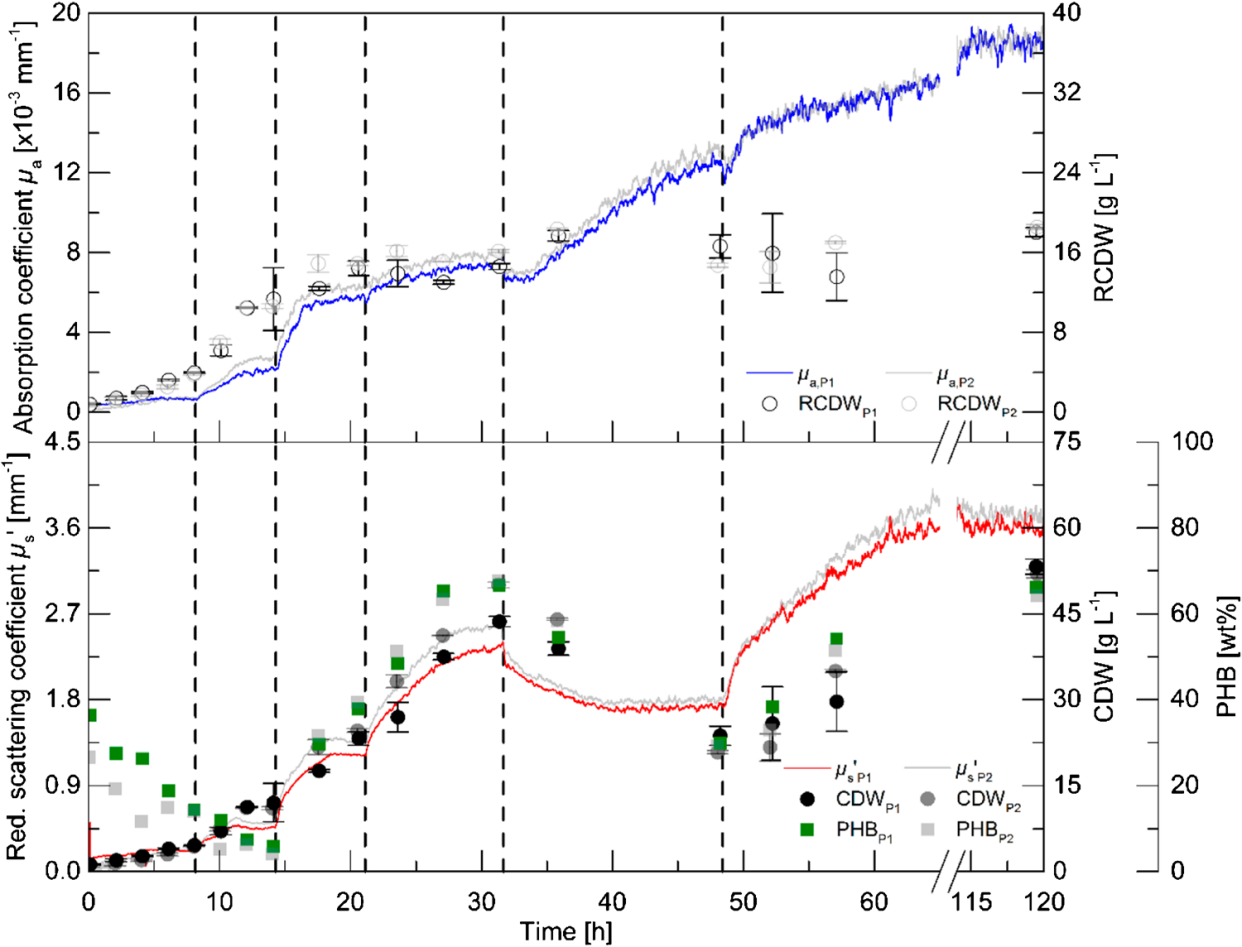
Bioreactor cultivation of *R. eutropha* in biological duplicate cultivation (blue, red, original; grey, replicate cultivation). Increasing *μ*_a_ and *μ*_s_′: growth phase with carbon and nitrogen in excess (1–15 h). Stagnation of *μ*_a_ and increasing *μ*_s_′: PHA formation phase with carbon in excess and depleted nitrogen (15–32 h). Increasing *μ*_a_ and decreasing *μ*_s_′: PHA degradation phase with depleted carbon and nitrogen in excess (32–49 h). Process end indicated by stagnation of *μ*_a_ and *μ*_s_′ (approx. 60 h). From [50]

Polyhydroxyalkanoates (PHAs) are biodegradable polymers produced as a carbon storage by microorganisms, such as *Ralstonia eutropha*, under nitrogen-limiting conditions with carbon available in excess. PHA accumulating microorganisms are composed of intracellular PHA inclusions called granules and the residual cell mass, which is bio-catalytically active. Similar to the *E. coli* results, it was shown that *μ*_s_′ correlates well with the whole biomass (PHA and residual cell mass together), while *μ*_a_ could be correlated with the accumulation of only the residual cell mass. Thus, PHA accumulation can be followed in real time. Different process states were identified in real time by *μ*_a_ and *μ*_s_′: growth phase with carbon and nitrogen in excess (increasing *μ*_a_ and *μ*_s_′), PHA formation phase with carbon in excess and depleted nitrogen (stagnation of *μ*_a_ and increasing *μ*_s_′), PHA degradation phase with depleted carbon and nitrogen in excess (increasing *μ*_a_ and decreasing *μ*_s_′), process end with depletion of the carbon and nitrogen source (stagnation of *μ*_a_ and *μ*_s_′) [50, 52] (Fig. 13). A duplicate cultivation yielding an almost identical result demonstrated the accuracy of the measurement method.

Microalgae cultivation in photobioreactors is crucial for the biofuel, nutraceutical, and bioproduct industries, with species like *S*. *rubescens* favored for their high growth rates. Sandmann et al. [51] investigated the use of PDW spectroscopy for inline monitoring of high-cell-density cultivation of *S*. *rubescens*. The experiments were conducted in a mesh ultra-thin layer photobioreactor, occupying a footprint of 4.84 m^2^. PDW spectroscopy was integrated into a recirculation loop of the reactor, allowing continuous measurement of biomass concentration over 3 weeks. The study demonstrated a strong correlation between reduced scattering coefficients and biomass concentration, confirming its suitability for real-time monitoring. Growth rates were successfully measured inline as well as nutrient depletion and dilution effects, providing superior temporal resolution compared to conventional offline methods such as dry matter content analysis and Coulter counter measurements. These findings highlight PDW spectroscopy as a valuable PAT tool for optimizing high-density algal cultivations.

### Cosmetics — emulsification

**Fig. 14:**
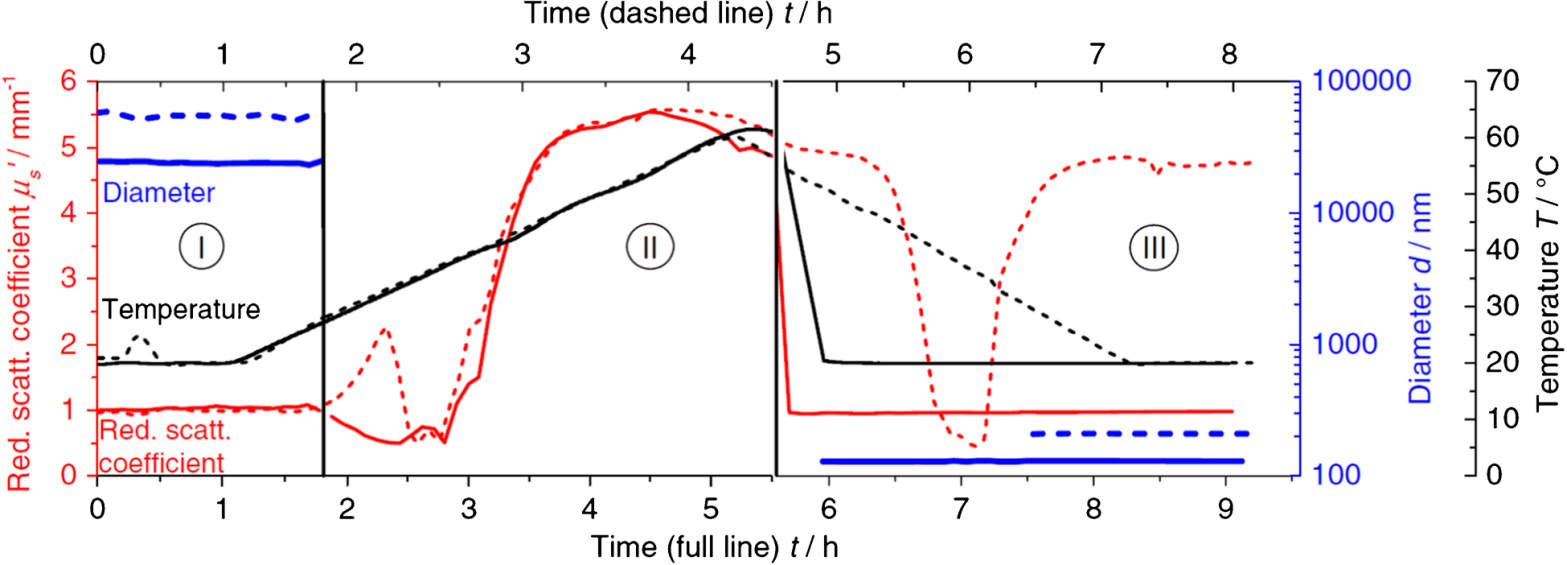
Phase inversion temperature emulsification of a 20 vol% water/Brij30/isohexadecane mixture for two different temperature profiles (solid and dashed line) and resulting droplet size and optical properties from PDW spectroscopy. From [55]

PDW spectroscopy was used to monitor an emulsification process of a water/Brij30/isohexadecane mixture at 20 vol% applying the phase inversion temperature method [45, 55]. This system is used in cosmetics as a model emulsion system to study formulations, mimicking oil-in-water emulsions commonly found in skincare products. The initial oil-in-water emulsion with droplet sizes of approx. 30 μm was processed into a water-in-oil nanoemulsion with droplet sizes of 100–200 nm by applying a defined heating and cooling profile. Different cooling rates influence final droplet sizes, which were monitored by PDW spectroscopy (Fig. 14). It was found that faster cooling generates smaller droplets. In addition, after nanoemulsion formation, inline Ostwald ripening rates were determined by continuous monitoring of nanodroplet growth.

## Conclusion and outlook

Over the past 25 years, photon density wave (PDW) spectroscopy has evolved from a proof-of-concept experiment into a robust and calibration-free process analytical technology (PAT) for the processing industries. Early limitations like the absence of dedicated instrumentation, process probes, and analysis software have largely been overcome. Current implementations deliver absolute optical coefficients and real-time, inline particle size information, features that are particularly valuable for monitoring industrial processes.

For concentrated liquid suspensions and emulsions, PDW spectroscopy is a powerful optical characterization technique, since it allows for the quantitative and calibration-free determination of the absolute optical coefficients of the material of interest. Here, the main advantage is the independent measurement of the light scattering and light absorption properties. In addition, based on theories of light scattering, particle or droplet sizes can be determined in real time without dilution. One fundamental difference and advantage compared to other sizing technologies lie in its theoretical foundation, which specifically addresses concentrated, multiple light scattering materials.

Good temporal resolution in combination with a probe-based measurement setup makes PDW spectroscopy well suited for process analytics in the process industry, with applications ranging from food, chemistry, pharma, biotechnology, to the cosmetic industry. Since it is entirely based on first principles, it eliminates the need for the often-found laborious model development and model maintenance work required by other optical PAT solutions, such as Raman or NIR spectroscopy.

However, in the world of PAT, it is still a comparably new technology and further developments will stimulate its applicability in industrial manufacturing. While it is currently well suited for implementation in R&D and pilot scale environments, further development of the PDW process probe technology, in particular, will help to bridge the gap towards broader industrial applicability. First and foremost, certification with respect to temperature, pressure, and pH is still required. Specific industrial segment demands like surface quality, material certification, or compliant wetted materials will need to follow. Other technical improvements should also address probe multiplexing, allowing one spectrometer to serve multiple process probes.

Regarding inline particle sizing, as an ensemble measurement method, PDW spectroscopy enables robust determination of mean particle sizes. For the determination of size distributions, however, PDW spectroscopy continues to rely on assumed size distributions like Gaussian or log-normal functions fitted to the experimental findings of the optical coefficients. Further research is required into the extension of multiple scattering theory to account for more complex particle size distributions.

PDW spectroscopy requires multiple light scattering and therefore high turbidity. However, a multitude of industrially relevant processes have been investigated, where significant turbidity is only observed at a later process stage. While the theoretical background of PDW spectroscopy is not suitable for very low turbidities, the spectrometer itself is extremely sensitive to changes in turbidities even under conditions without multiple light scattering. To overcome this gap of missing information, an add-on raw data analysis (“PDraW”) has recently been implemented to additionally provide process information during regimes where the PDW theory is not applicable.

From a higher level perspective, future developments might result in hybrid PAT approaches that combine PDW spectroscopy with complementary optical methods, such as Raman or NIR spectroscopy. Including information from processing parameters like mass flow or density, this holistic processing data might enhance process understanding by providing particle, physical, and chemical information simultaneously. Such integrations could enable robust model-predictive control strategies and open new pathways for the application of PDW spectroscopy and other PAT in advanced manufacturing processes.

Novel materials that have already been investigated with PDW spectroscopy include nanoparticles and biodegradable plastics, highlighting their versatility for both nanostructured and sustainable systems. Looking ahead, promising applications of novel materials could be precursor materials for battery production, where particle size is crucial for product quality. In the field of pharmaceutical biotechnology, PDW spectroscopy might be applied to track cell sizes and the formation of inclusion bodies in microorganisms, such as those occurring in industrial insulin production.

There is strong potential for this technology to find more and more applications in the challenging world of heterophase material processing. Its distinctive features, such as the absolute, calibration-free measurement of optical coefficients and real-time inline particle sizing, may support more resource-efficient, automated, and quality-controlled manufacturing processes.
